# Prevention of a Parastomal Hernia by Biological Mesh Reinforcement

**DOI:** 10.3389/fsurg.2015.00053

**Published:** 2015-10-22

**Authors:** René H. Fortelny, Anna Hofmann, Christopher May, Ferdinand Köckerling, BioMesh Study Group

**Affiliations:** ^1^Department of General, Visceral and Oncological Surgery, Wilhelminenspital, Vienna, Austria; ^2^Department of Surgery and Center for Minimally Invasive Surgery, Vivantes Hospital, Berlin, Germany

**Keywords:** parastomal hernia, parastomal hernia repair, parastomal hernia prevention, biologic mesh, bio mesh, bio-prostethic mesh

## Abstract

**Introduction:**

In the field of hernia prevention, the prophylactic mesh-reinforcement of stoma-sites is one of the most controversially discussed issues. The incidence of parastomal hernias in the literature reported to be up to 48.1% after end colostomy and up to 30.8% after loop of colostomy, but still remains uncertain due to diagnostic variety of clinical or radiological methods, heterogeneous patient groups and variable follow-up intervals. Anyway, the published data regarding the use of synthetic or bio-prostethic meshes in the prevention of parastomal hernia at the primary operation are very scarce.

**Methods:**

A literature search of the Medline database in terms of biological prophylactic mesh implantation in stoma creation identified six systematic reviews, two randomized controlled trials (RCT), two case-controlled studies, and one technical report.

**Results:**

In a systematic review focusing on the prevention of parastomal hernia including only RCTs encompassing one RCT using bio-prosthetic mesh the incidence of herniation was 12.5% compared to 53% in the control group (*p* < 0.0001). In one RCT and two case-control studies, respectively, there was a significant smaller incidence of parastomal herniation as well as a similar complication rate compared to the control group. Only in one RCT, no significant difference regarding the incidence of parastomal hernia was reported with comparable complication rates.

**Conclusion:**

Thus, so far two RCT and two case-control studies are published with prophylactic bio-prosthetic reinforcement in stoma sites. The majority revealed significant better results in terms of parastomal herniation and without any mesh-related complications in comparison to the non mesh group. Further, multicenter RCT are required to achieve a sufficient level of recommendation.

## Introduction

The BioMesh Study Group has set itself the task of identifying the best way to use biological meshes for various indications. The first step toward achieving that goal is to compile systematic reviews of the different indications on the basis of the existing literature. The available literature sources will be evaluated in accordance with the Oxford Centre for Evidence-based Medicine-Levels of Evidence (March 2009). Next, based on the review findings, corresponding Statements and Recommendations are to be formulated in a Consensus Conference for the use of biological meshes for the different indications. The findings of the Consensus Conference are then to be summarized for a joint publication. This present publication is part of the project undertaken by the BioMesh Study Group.

In the field of hernia prevention, the prophylactic mesh-reinforcement of stoma-sites is one of the most controversially discussed issues. The exact incidence of parastomal hernias remains uncertain due to diagnostic variety of clinical or radiological methods like ultrasound and computed tomography, heterogeneous patient groups, and variable follow-up intervals (1). Based on a meta-analysis by Carne et al. (2), the incidence for parastomal hernia ranges from 1.8 to 28.3% for end ileostomies and 0–6.2% for loop ileostomies. In case of end colostomy, the hernia rates are reported as 4.0–48.1% and in case of loop colostomy, the hernia rates are 0–30.8% after 10-year follow up. In a life time analysis of stomal complications such as bulge, abdominal discomfort, abdominal pain, constipation, incarceration, ileus, and parastomal herniation following colostomy can be occur in a time frame of up to 20 years postoperatively (3). It seems that one of the most successful prevention of stoma site hernias is the use of a prophylactic mesh. The risk of colostomy herniation seems to be doubled in comparison to an ileostomy. The relation of a larger diameter of the trephine to the abdominal wall defect in case of colostomy creation might be the main reason. There are different surgical options for parastomal hernia repair. In the current review of Aquina et al. (4) the cumulative recurrence rates in the literature for open surgery are reported to be 67.6% after suture repair, 18.2%, after mesh only repair and 9.6% after retromuscular mesh repair. For laparoscopic surgery recurrence rates were 30% after mesh repair by keyhole technique, 8.1% by Sugarbaker technique and 2.1% after sandwich technique respectively. In another review concerning the use of biologic grafts for parastomal hernia repair by Slater et al. (5), four retrospective studies (combined enrollment of 57 patients) obtained a cumulative recurrence rate 15.7% [95% confidence interval (CI) 7.8–25.9] and wound-related complications in 26.2% (95% CI 14.7–39.5). No mortality or graft infections were reported.

But anyhow following questions still remain: first, the selection of mesh type and location at the primary operation for the prevention of hernia development and second is there any indication for the use of bio mesh in a clean contaminated field.

## Methods

A literature search of the Medline database using the PubMed search engine, using the keywords (parastomal hernia OR parastomal hernia repair OR parastomal hernia prevention AND biologic mesh AND biomesh AND bio mesh) returned 236 hits up to June 2015. Titles and abstracts were scrutinized on the use of prophylactic biologic mesh reinforcement of the stoma site at the primary operation. The full text of 25 articles was assessed and 11 relevant papers were identified including six systematic reviews (4, 6–10), two randomized controlled studies (RCT) (11, 12), two case-controlled studies (13, 14), and one technical report (14). A summary of study characteristics and outcomes is presented in Table 1. Qualitative assessment of all included studies was performed using the Oxford Centre for Evidence-Based Medicine 2009 levels of evidence.

**Table 1:** **Studies of prevention of parastomal hernia**.

Author	Study design	Patient (*n*) (mesh vs. control)	Mean age/BMI (mesh vs. control)	Stoma type	Mesh type	Mesh size/mesh position	Fixation	Follow up in month (mesh vs. control)	Herniation(mesh vs. control)	Infection (mesh vs. control)	LoE
Williams et al. (14)	Pilot CS	33 (22 vs. 11)	49 vs. 59	Ileostomy, colostomy	Permacol	7 cm diameter onlay	sutures	18 (10–24) vs. 9 (4–25)	3 vs. 8	0	2b
			33 vs. 29								
Fleshman et al. (12)	Multicenter RCT	113 (55 vs. 58)	60.25 vs. 59.1	Ileostomy, colostomy	Strattice	4.8 cm × 4.8 cm sublay	No	24	6 vs. 7	3 vs. 2	1b
			26.6 vs. 24.7								
Figel et al. (13)	Retrospective CS	16 (no controls)	63.1	End stoma	Surgisis EXL	13 cm × 22 cm sugarbaker (75%) and keyhole (25%)	NR	38 (24–53)	0	NR	3b
			11 patients > 30								
Hammond et al. (11)	RCT	20 (10 vs. 10)	42.6 vs. 50	Defunctioning loop stoma	Permacol	10 cm × 10 cm pre-peritoneal	sutures	12	0 vs. 3	0	1b
			26.3 vs. 26.3								

*n, number; CS, Case-controlled study; RCT, Randomized Controlled Trial; LoE, Level of Evidence; Permacol, porcine-derived, cross-linked acellular dermal matrix; Strattice, porcine-derived, non-cross-linked acellular dermal matrix; Surgisis EXL, porcine-derived, non-cross-linked submucosa matrix; NR, no report*.

## Results

The reviews of Aquina et al., Hotouras et al., Shabbir et al., Sajid et al., Wijeyekoon et al., and Tam et al. (4, 6–10) all focused on parastomal hernia prevention by the placement of a mesh (synthetic and biological) at the primary operation. Aquina et al. (4) reported a cumulative incidence of parastomal hernia rate of 10.7% including the RCT of Hammond et al. (11) using a biologic mesh (Permacol). In the systematic review of Shabbir et al. (7), three RCT [Hammond et al., Jänis et al., and Serra-Aracil et al. (11, 15, 16)] were enclosed. The analysis of the three RCT comprising a total of 128 patients (64 with mesh, 64 without mesh) revealed a hernia incidence of 12.5% compared to 53% in the control group [risk ratio, 95%, CI, 0.25 (0.13, 0.48), *p* < 0.0001] in a follow up period of 7–83 months. Two of the studies (11, 16) used clinical and radiological examinations. Concerning mesh-related morbidity, no difference was detected. The systematic review of Sajid et al., Wijeyekoon et al., and Tam et al. (7–9) all including the same RCT (11, 15, 16) obtained identical results.

In 2008, the first RCT focusing on the use of biological mesh for parastomal hernia prevention was published by Hammond et al. (11). Twenty patients undergoing a defunctioning stoma operation were randomized to an interventional group with reinforcement by porcine-derived, acellular dermal sheet, cross-linked acellular dermal sheet (Permacol, Tissue science laboratories, Aldershot, Hants, UK) or a conventional group without mesh. The trephine of the abdominal wall including the rectus sheath was defined by 2 cm × 2 cm. The biological mesh measuring 10 cm × 10 cm was supplied with a center keyhole of 2 cm and positioned between posterior layer of the rectus sheath and the peritoneal membrane – described as pre-peritoneal position and fixated by interrupted 3/0 prolene sutures to the rectus sheath by an inner and outer suturing at four positions.

The patient controls were performed at the time of stoma reversal or in cases of non-stoma reversal, at 12 months. At a median follow up of 6.5 months, three patients suffered from a parastomal hernia in the control group and no patient in the treatment group. Stoma site ultrasound assessment was performed in 7 of 10 patients in the treatment group and 9 of 10 in the control group. There were no detected differences concerning the infection signs or other complications. The shortcomings of this randomized controlled phase 1 study are the low number of patients enrolled, the short follow-up period and an unexpected very high percentage of stoma site hernias in the control group in comparison to the published literature (2).

The second study selected in this review published in 2012 is a retrospective case-control study by Figel et al. (13). A biologic mesh derived from porcine submucosa (Surgisis EXL, Cook Surgical, Bloomington, IN, USA) and non-cross-linked with a size of 13 cm × 22 cm was placed at the time of creation of an intestinal end stoma in a Sugarbaker position (12 patients) and in keyhole-technique (four patients). Sixteen patients were enrolled. No mesh related complications and no parastomal hernias were detected in a median follow-up of 38 months. This study confirmed the safety, efficacy, and cost-effectiveness, respectively, of prophylactic bio-prosthetic mesh reinforcement at the time-point of permanent stoma creation.

In the year 2014, another prospective, multicenter, randomized, controlled, double-blinded study of non-cross-linked porcine acellular dermal matrix (PADM; Strattice, LifeCell Corporation, Branchburg, NJ) in patients undergoing elective surgery for permanent end stoma (71 colostomies, 42 ileostomies) was published by Fleshman et al. (12). Fifty-five patients were treated with the use of PADM in a size of 6 cm × 6 cm or 8 cm × 8 cm (median size after trimming 4.8 cm × 4.8 cm) with a cruciate incision of 2 cm for the bowel passage (incision was enlarged in 78.2%) in a retro-muscular sublay position using no fixation. The control group consisted of 58 patients without mesh reinforcement. Intraoperative complications, blood loss, and quality of life-scores were without significant differences in either group. The postoperative investigations were performed by a blinded assessor and an abdominal CT (11 patients) was followed in case of suspected herniation at the stoma site. The incidence of parastomal hernias in a follow up of 24 months was 12.2% in the PADM-group and 13.2% in the controls without significant difference. The ostomy circumference in the PADM group was significant larger (6.4 ± 3.9 vs. 4.8 ± 2.9 cm; *p* = 0.002) compared to the control group, which may be a predisposition for the development of a parastomal hernia and represents a potential bias of the study. In a letter to the editor, Hontouras (17) discussed the important role and risk of oversized stoma aperture for the development of a hernia. Based on the study of Pilgrim et al. (18), a stoma aperture >35 mm is an independent risk factor for hernia development – increasing by 10% for every millimeter increase in size. In summary, the RCT of Fleshmann et al. confirms the safety of prophylactic biological reinforcement. However, based on the results of parastomal hernia incidence in comparison to the control group, no recommendation for the use of bio-prosthesis can be given.

Recently published in 2015, Williams et al. (14) reported a case-controlled pilot study based on a stapled mesh stoma reinforcement technique (SMART), which was already introduced in 2011 (19). A special designed circular stapler gun (Compact™, Frankemann International Limited) was used in combination with a porcine-derived cross-linked acellular dermal sheet (Permacol™, Covidien plc, 20 Lower Hatch St, Dublin 2, Ireland), which is configured in a circular design with a diameter of 7 cm. After excising, a cylinder of abdominal wall and subcutaneous fat a cruciate incision of the rectus sheath is performed. The knife diameter used (17, 21, or 24 mm) is dependent on the diameter of the bowel used to be traverse the stoma trephine. The shaft of the anvil is delivered through the posterior rectus sheath and mated with the trocar of the circular stapling device after preloading with the mesh. The circular stapling device is closed, fired, and removed, encompassing a disc of mesh, posterior rectus sheath and peritoneum and leaving a precise reinforced stapled trephine. Finally, the outer mesh circumference is sutured to the anterior rectus sheath. Twentytwo patients were included and received stoma formation with SMART-technique and another 11 were assigned to the control group without reinforcement of the stoma site (18 open: 4 laparoscopic; 11 ileostomies: 11 colostomies). All SMART stomas were fashioned using a circular stapler with a 24-mm knife diameter. Patients with either complications from a pre-existing stoma (*n* = 15) – large parastomal hernia unsuitable for local repair (*n* = 6) or recurrent herniation as a result of previous repair (*n* = 9) or underlying conditions (*n* = 7) – such as obesity, asthma, corticosteroid use, collagen disorder or combination of these respectively underwent a resiting SMART-procedure at the index operation.

There were no intraoperative or early stoma complications. Recurrent parastomal herniation was diagnosed in four patients (19%) of the SMART group, which was significantly lower (*p* = 0.003) in comparison to 8 patients (73%) in the control group. Designed as a pilot study, there are some basic limitations, such as missing randomization, heterogeneity of patients and short follow up. But this new technique could be promising in high risk patients and the results of an ongoing randomized trial (ISRCTN 94943190) in this technique should be give us more detailed information and conclusions.

Another ongoing multicenter RCT from France (Centre Hospitalier Universitaire, Amiens) comparing prophylactic biological mesh vs. no mesh in colorectal surgery with colostomy (“Prospective, Multicenter, Randomized, Parallel Group Clinical Study Evaluating the Efficacy of a Biological Mesh (Strattice™) for the Prevention of Parastomal Hernia After Colorectal Surgery With Colostomy,” NCT02121743), should be completed by April 2016.

## Discussion/Summary

The current literature supports the significant risk reduction of parastomal hernia development by mesh reinforcement of the permanent stoma at the primary operation. Based on the published literature, the prophylactic mesh application is not associated with a significant increase of mesh-specific complications and comorbidities such as seroma, infection and migration. Concerning the choice of mesh, synthetic or biologic prosthesis, there are only four level 1 b studies – two with the use of synthetic meshes in retro-muscular position (15, 16) as well as two with biologic mesh reinforcement in sublay position (11, 12). The best option of mesh placement – onlay, sublay, or intraperitoneal – keyhole, sugarbaker, sandwich, or by a 3D funnel mesh type (20) – remains unclear and has to be compared in further RCT. In summary, so far now only two RCT (11, 12) and two case-control studies with prophylactic biomesh reinforcement in stoma sites are published. Both studies have to be looked at very critically due to limitations [too small numbers of patients and short follow up (11)] and a heterogeneity of patients regarding the different trephine sizes to the abdominal wall (12). However, in both RCT as well in the two case-control studies (13, 14), no bio-mesh related complication was observed. The discussion addressing the topic of crosslinking vs. non-crosslinking in terms of susceptibility to infection and failure of remodeling (bulging) in this special indication remains unclear, since we do not have any late term results and both studies used different bio-prosthesis (Permacol™, Surgisis™).

Nevertheless, we have to consider that only 50% of patients will develop a parastomal hernia by using non-mesh techniques and there is a risk of overtreatment if all patients receive a prophylactic mesh. So, it is mandatory to investigate which patients are at a significant risk of developing a parastomal hernia. In conclusion, it seems to be beneficial to place a mesh at the primary operation when performing a permanent stoma based on the available literature, which describes no increase of complications, easy performance, and cost-effectiveness (13).

In summary, based on the data available, the prophylactic placement of mesh at the index operation associated with stoma creation needs scientific attention in the near future.

The remaining questions concerning the choice of mesh material, mesh design, and most favorable anatomical location for the mesh have to be answered by additional well-designed prospective multicenter studies.

## Conflict of Interest Statement

The authors declare that the research was conducted in the absence of any commercial or financial relationships that could be construed as a potential conflict of interest.

## Appendix

BioMesh Study Group:

Ferdinand Köckerling (Chairman), Stavros Antoniou, René H. Fortelny, Frank A. Granderath, Markus Heiss, Franz Mayer, Marc Miserez, Agneta Montgomery, Salvador Morales-Conde, Filip Muysoms, Alexander Petter-Puchner, Rudolph Pointner, Neil Smart, Marciej Smietanski, Bernd Stechemesser.
